# A Systematic Review on Materno-Foetal Outcomes in Pregnant Women with IgA Nephropathy: A Case of “Late-Maternal” Preeclampsia?

**DOI:** 10.3390/jcm7080212

**Published:** 2018-08-11

**Authors:** Giorgina Barbara Piccoli, Isabelle Annemijn Kooij, Rossella Attini, Benedetta Montersino, Federica Fassio, Martina Gerbino, Marilisa Biolcati, Gianfranca Cabiddu, Elisabetta Versino, Tullia Todros

**Affiliations:** 1 Dipartimento di Scienze Cliniche e Biologiche, Università di Torino, 10100 Torino, Italy; 2 Nephrologie, Centre Hospitalier Le Mans, Avenue Roubillard, 72000 Le Mans, France; 3 Unità Materno-Fetale, Dipartimento di Chirurgia, Università di Torino, 10100 Torino, Italy; isabelle.kooij@hotmail.com (I.A.K.); rossella.attini@gmail.com (R.A.); benedettamontersino@yahoo.it (B.M.); federica.fassio@hotmail.it (F.F.); martigerby@hotmail.it (M.G.); marilisa.biolcati@unito.it (M.B.); tullia.todros@unito.it (T.T.); 4 Nefrologia, Ospedale Brotzu, 09100 Cagliari, Italy; gianfranca.cabiddu@tin.it; 5 Epidemiologia, Dipartimento di Scienze Cliniche e Biologiche, Università di Torino, 10100 Torino, Italy; elisabetta.versino@unito.it

**Keywords:** IgA nephropathy, pregnancy, preeclampsia, proteinuria, preterm delivery, systematic review

## Abstract

Background: IgA nephropathy is the most common primary glomerulonephritis in pregnancy and shares with other immunologic diseases and kidney diseases a relationship with adverse maternal outcomes, whose entity and pattern is only partially quantified. Recent studies provide new information and a systematic review regarded progression of kidney disease. The discussion of the outcomes with respect to low-risk pregnancies may help to perfect the estimation of the risks, and to identify specific research needs. Methods: A search strategy was built on Medline, EMBASE and the Cochrane review for the period January 2000–April 2017, aimed at retrieving both case series (defined as with at least 6 pregnancies in women with IgA nephropathy) and case reports, to look into rare occurrences. All papers, with or without control groups, were selected if they reported on at least one pregnancy outcome, or on long-term kidney function. Search strategy, paper selection and data extraction were done in duplicate (PROSPERO N 42016042623). Meta-analysis of case series was performed with Metanalyst Beta 3.13. Case reports were analysed narratively. Results: The search retrieved 556 papers, of which 27 were included (13 series and 14 case-reports). The case series report on 581 women with 729 pregnancies. The analysis was performed in comparison to the available control groups: 562 non-pregnant controls were available for the analysis of progression of kidney disease. As for pregnancy related outcomes (preeclampsia (PE), pregnancy induced hypertension (PIH), preterm birth, small babies), we meta-analyzed the data with respect to the only series of low-risk pregnancies (1418 pregnancies). When compared with women who never got pregnant after diagnosis of IgA nephropathy, in the present meta-analysis pregnancy in women with IgA nephropathy was not associated with a higher risk of progression of kidney disease, possibly due to the overall preserved kidney function at baseline: end-stage kidney disease (OR 0.68; CI 0.28–1.65). Conversely, the incidence of adverse pregnancy-related outcomes was increased compared to low-risk controls: PE and PIH were more than ten-fold increased (OR 11.80; CI 7.53–18.48 and OR 10.39; CI 5.45–19.80), while the increase in risk of preterm birth and “low birth weight babies” was less marked (OR 3.37; CI 1.91–5.95 and OR 2.36; CI 1.52–3.66), a discrepancy suggesting the occurrence of “late” or “maternal” PE, that may affect less severely foetal growth or shorten gestation. In conclusion, in the present meta-analysis IgA nephropathy was not associated with an increased progression of kidney disease. The more than ten-fold increased risk of PIH and PE, in combination with a doubled risk of small babies, suggests the occurrence of “late” or “maternal” PE, usually less affecting early foetal growth. This finding may be of help in defining control policies, while further research is needed to guide clinical management.

## 1. Introduction

IgA nephropathy is probably the most common primary glomerular nephritis worldwide; its higher incidence in young people makes it highly relevant in pregnancy [1].

The present term of IgA nephropathy encompasses two previously defined diseases, usually known by their eponyms: Berger’s disease, in which the IgA deposition is limited to the kidney, and Henoch-Shönlein, in which IgA nephropathy is a part of a systemic vasculitis that could involve the skin and the gastro-intestinal tract, with asymmetric acute osteoarthritis [2,3]. The recognition of family clustering of the two diseases, and the identical renal pathology led to gather both diseases under a unifying definition [4,5,6]. IgA nephropathy may present with a wide spectrum of clinical presentations, from mild, recurrent and remittent microhematuria to boosts of macrohematuria and nephritic syndrome, or a severe and rapidly progressive disease [1,4,7,8]. Hypertension and week are considered as negative outcome predictors, even if only mild. Their negative effect on outcome is observed even in the absence of reduced renal function, which is a likewise (albeit tautological) acknowledged progression marker itself. Other histological markers of disease progression and risk are still subject of debate [7,8,9,10,11,12,13,14]. The risk of progression to end stage renal disease (ESRD) has been differently evaluated. However, this risk is not negligible over the entire life span as progression to ERSD is reported from rare to frequent, depending on morphological, clinical and laboratory characteristics, and treatment. For these reasons, IgA nephropathy is usually considered as a disease that may remit, but that is never completely cured [14,15,16,17,18]. 

The acknowledgement of the importance of early renal disease in pregnancy is particularly relevant for IgA nephropathy, due to its relatively high prevalence in young age, slow progression, with frequent preservation of the kidney function at least for long periods, making normal kidney function common in affected patients in childbearing age, and heterogeneous presentation, that may impair early diagnosis [19,20,21,22]. 

As will be further discussed in detail, a systematic review, encompassing a wide time span, was recently focused on the progression of chronic kidney disease in patients with IgA nephropathy who had been pregnant [23]. To try to further add to the knowledge in this important field, we considered several recent publications, not available at the time of the first review, and we focused a second systematic review on the effect of IgA nephropathy on pregnancy and of pregnancy on the progression of IgA nephropathy, on the new millennium, on the account of the changes in disease management and maternofetal care occurring over time, and we attempted for the first time a meta-analytical comparison with a low-risk population, in order to better quantify the risk of the various adverse pregnancy outcomes, as a further support for counseling.

## 2. Methods

### 2.1. Eligibility Criteria

The eligibility criteria were broad, because of the expected retrieval of heterogeneous designs and definitions. Consequently, we included all published studies that dealt with IgA nephropathy in pregnancy and that reported on at least one pregnancy-related outcome, or on long-term kidney function. We included all study designs, i.e., prospective or retrospective cohort studies, case-control studies, trial-based analyses, and case reports. Reviews, whether or not systematic, were also gathered to check for papers eventually escaped from our wide search strategy. The search was not limited to papers which provided a control group, but information on all control groups were gathered. 

Studies were divided into those regarding more than 5 or less than 6 cases (defined as case series and cases reports, respectively). 

The review was prospectively registered on PROSPERO International prospective register of systematic reviews (N 42016042623).

The review was performed according to the Meta-analysis of observational studies in epidemiology (MOOSE) criteria. 

### 2.2. Search Strategy

We searched MEDLINE, PubMed, Embase and the Cochrane Review database from January 2000 to April 30th 2017 using a combination of MeSH terms and keywords related to IgA nephropathy and pregnancy, including: pregnancy, pregnant, gestation* and IgA nephropathy, Henoch* or Shoenlein*, Berger*. We also checked the reference lists of relevant articles and citations of included studies.

We did not limit our search to any language, but we limited it to the period starting from 2000, allowing for the considerable changes in management of the disease and in maternofetal follow-up that occurred in the new millennium, in order to implement the clinical decision rules presently recommended by the very few guidelines or consensus statements dealing with glomerular diseases in pregnancy [24,25,26]. 

All articles identified were screened for eligibility on the basis of the content only: all papers reporting on any form of IgA nephropathy in pregnancy and reporting on any outcome were selected. The search was performed by G.B.P. and I.A.K., after which I.A.K. and R.A. did a first screening of the papers. The final selection was agreed between G.B.P. and I.A.K., any discrepancy was resolved by discussion.

### 2.3. Data Extraction and Quality Assessment 

Two reviewers (I.A.K. and G.B.P.) extracted study and population characteristics and outcomes. A third reviewer (G.C.) double-checked the accuracy of data entered from each study in case of discrepancies. All definitions of IgA nephropathy, pre-eclampsia (PE), pregnancy induced hypertension (PIH), small babies, small for gestational age babies (SGA) or other pregnancy related outcomes were gathered.

Due to the lack of randomized controlled trials (RCTs), and to the limited number and type of control groups, no formal analysis of risk of bias was performed. No selection for quality was performed. However, the studies were critically analyzed and the completeness of the data was considered as an indirect marker of quality.

The case series were compared with the controls available, regarding progression of chronic kidney disease (CKD), and with the only low risk population available, regarding the various pregnancy outcomes. Odds ratios and confidence intervals were calculated (OR, CI). The case reports were described narratively. 

### 2.4. Data Synthesis

The PICOS criteria were adapted to this particular situation in which pregnancy is considered the “intervention” as follows: 

P—Patients: women with IgA nephropathy in pregnancy; 

I—Intervention: in the absence of a true “intervention”, pregnancy was considered as the “intervention”;

C—Comparators: in the case of progression of kidney disease, non-pregnant IgA patients were considered as comparators. In the case of pregnancy outcomes, considered low-risk pregnancies or pregnancies in women not affected by IgA nephropathy (or other kidney diseases); 

O—Outcomes: progression of kidney disease; all the materno-fetal outcomes described in the papers (including, but not limited to, PE, PIH, preterm delivery, small babies, SGA). 

S—Studies: all studies, regardless of the study design, reporting on pregnancy outcomes or on long-term kidney function in patients with IgA nephropathy.

Statistical analysis was performed using Metanalyst Beta 3.13. 

Due to the high baseline heterogeneity a random effect model was employed. 

Stratification for baseline renal function, hypertension and week was attempted, but was not possible due to the high heterogeneity of the papers. 

The control groups retrieved mainly regarded progression of kidney disease in women with IgA nephropathy who did not get pregnant after diagnosis; these data were used to analyze the effect of pregnancy on kidney function. 

For pregnancy-related outcomes, we employed as a control group the only low-risk population available (from the cohort named TOCOS from the Torino Cagliari Observational Study) and meta-analyzed each study with respect to it.

## 3. Results

### 3.1. Case Series: Overall Data

**Figure 1:**
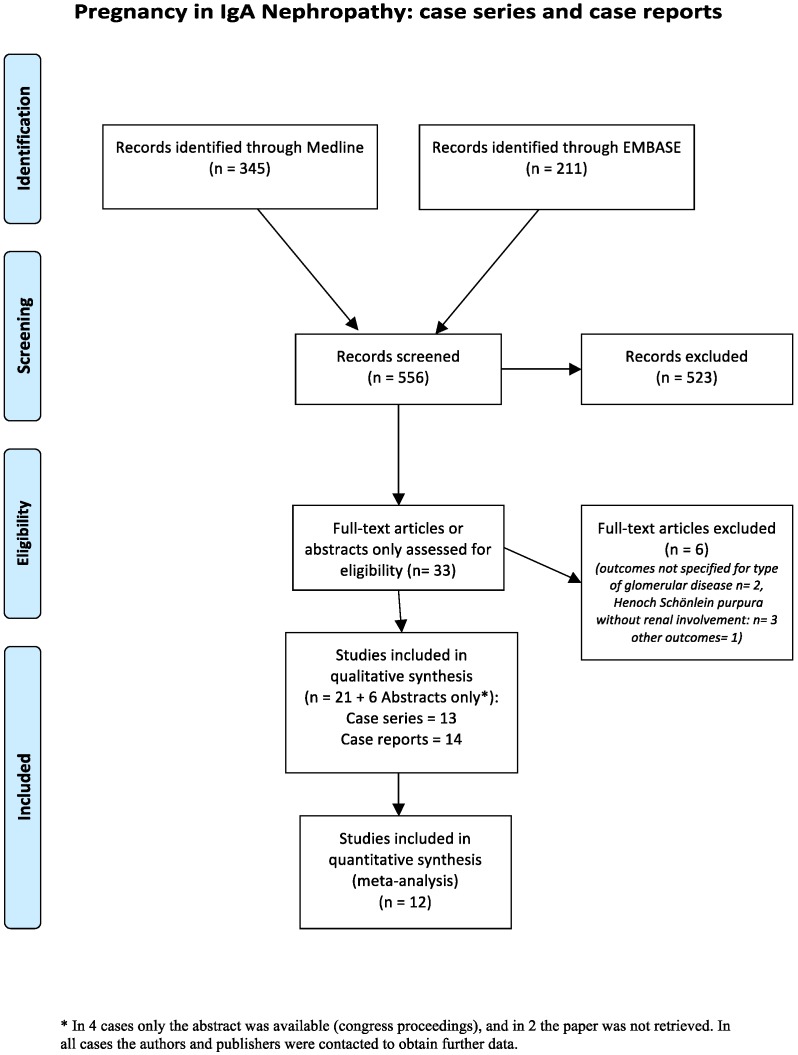
The flow-chart of the retrieved papers.

The search strategy identified 556 articles, of which 27 met the inclusion criteria: 13 case series, which we attempted to meta-analyze, for the various outcomes, and 14 case reports, discussed narratively (Figure 1) [27,28,29,30,31,32,33,34,35,36,37,38,39,40,41,42,43,44,45,46,47,48,49,50,51,52,53]. 

The main characteristics of the case series are reported in Table 1, Table 2, Table 3 and Table 4 [27,28,29,30,31,32,33,34,35,36,37,38,39].

Overall, the case series included four prospective studies, eight retrospective studies and two matched cohort studies, the latter regarding progression of CKD in women affected by IgA nephropathy, with or without pregnancy (Table 1). Globally, the studies report on 581 women with 729 pregnancies in IgA nephropathy (assuming one pregnancy per woman if not otherwise specified), and on 562 non-pregnant controls distributed over three studies; conversely, normal pregnancies were available in only one study [30].

The studies are heterogeneous for the number of pregnancies (12 to 229), setting of study (seven from Asia, five from Europe, one from the USA and one from Saudi Arabia), and study aims. Six studies provided information on the type and frequency of control policies in pregnancy and during follow-up (Table 2). 

### 3.2. Case Series: Pregnancy Outcomes 

Table 3 reports on the main pregnancy-related outcomes reported in the papers. Given the different study designs, the included studies reported on different outcomes: neonatal deaths were reported on by 4 studies, live births were reported on by 8 studies, preterm delivery was reported by 8, and need for admission to neonatal intensive care unit by one study only. All studies reported on preeclampsia (PE) and/or hypertensive disorders of pregnancy. Information on birth weight was available in most studies, but the information was not always contextualized to gestational age, and the information on neonates small for gestational age was available in two studies only.

Overall, the papers describe a population with high incidence of PIH (reported in around 30% of the cases, however, the distinction with preexisting hypertension is usually lacking, and this leads to a possible underestimation of new onset hypertension) and PE (reported in around 15% of the cases), with about 15% incidence of preterm deliveries, usually without exhaustive specification of gestational age (Table 3). 

Table 4 reports on baseline kidney function and other maternal outcomes: baseline kidney function (glomerular filtration rate: GFR) was relatively well preserved in most studies but was fully normal in 2 only (GFR was >100 mL/min in 2 studies and <100 mL/min in 7 studies). Mean week was around 1 g/day. Prevalence of hypertension was not clearly defined at baseline, and the distinction between previous use of angiotensin converting enzyme-inhibitors (ACEi) or Angiotensin II receptor blockers (ARBS) for week , hypertension or both was not available.

Four of five studies reporting on kidney function over time did not find any difference between cases and non-pregnant controls during follow-up, considering however different outcomes (start of dialysis, doubling of serum creatinine, GFR decrease, new-onset hypertension); the fifth, most recent study, found a correlation between kidney function worsening and the occurrence of adverse pregnancy outcomes [27] (Table 4).

### 3.3. Case Reports 

The main characteristics of the case reports are summarized in Table 5, Table 6 and Table 7 [40,41,42,43,44,45,46,47,48,49,50,51,52,53]. 

Keeping in mind the aim to discuss severe or unusual situations, the cases reported on various situations, including new onset-diagnosis of IgA nephropathy in pregnancy, recurrence of IgA nephropathy in a kidney allograft, severe reduction of the kidney function (5 cases); one case reported on three pregnancies in the same woman (one occurring in the pre-dialysis phase, two on dialysis) (Table 5). 

Presumably as a consequence of the different selection of the cases compared to that of the series, most of the babies were born preterm (61.5%); maternal complications were common (30%) and more than half of the newborns had low birth weight. Nevertheless, all reported babies had a favorable outcome. Conversely, dialysis was needed in pregnancy in three cases and half of the patients developed hypertension or preeclampsia. 

### 3.4. Meta-Analysis

#### Kidney Function

**Figure 2:**
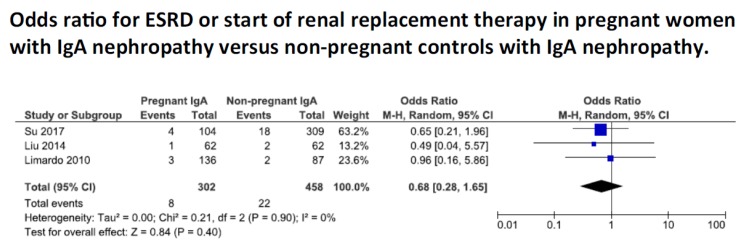
Odds ratio for end-stage renal disease in patients with IgA nephropathy, with or without pregnancy. Legend: ESRD: end stage renal disease. CI: confidence intervals.

Figure 2 reports on ESRD and the start of renal replacement therapy in patients with and without pregnancy. The incidence was low in the five studies reporting on this outcome: 11/330 patients versus 22/458 controls, and there was no significant difference found between the two groups (OR 0.68; CI 0.28–1.65). 

These reassuring data have to be contextualized to patients with good renal function, and it has to be taken into account that time of follow up differed notably between the studies (ranging from 1 to 10 years postpartum), which reduces the clinical value of this comparison (Figure 2).

### 3.5. Pregnancy Related Outcomes

**Figure 3:**
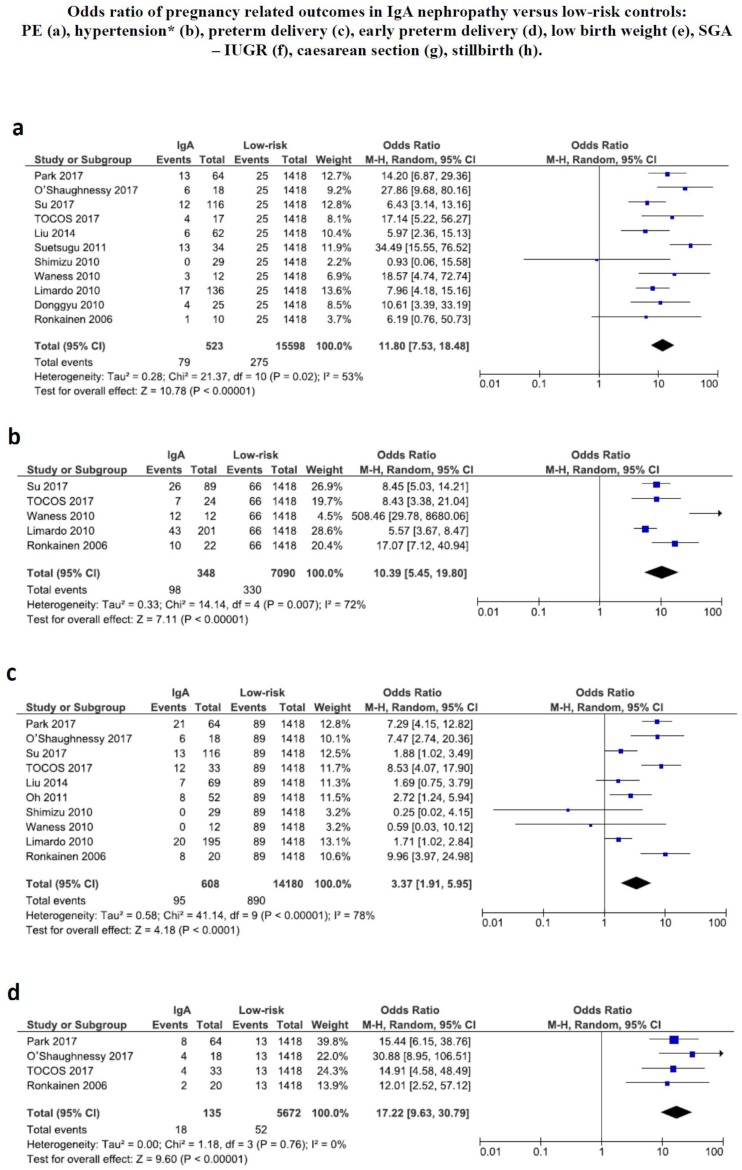

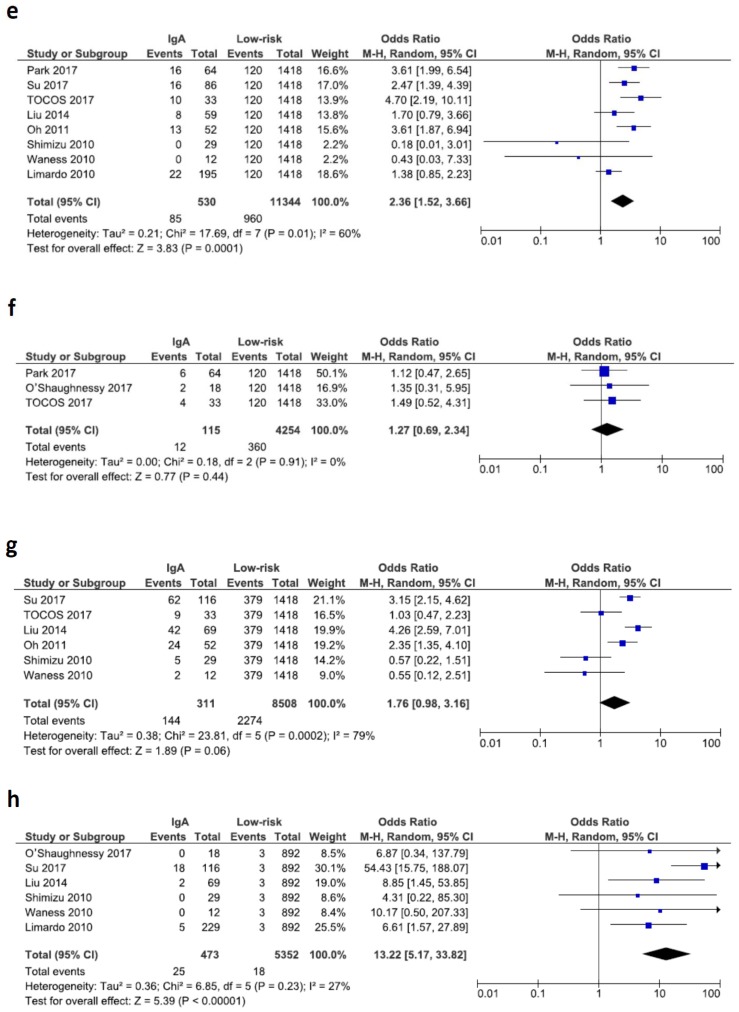
Odds ratio for different pregnancy related outcomes. SGA: small for gestational age. IUGR: intrauterine growth restriction. PE: preeclampsia. CI: confidence intervals.

The risk for adverse pregnancy-related outcomes was higher in IgA patients than in the low-risk cohort (Figure 3). 

While the incidence of caesarean sections and the incidence of small for gestational age babies were not significantly different in IgA nephropathy and in the low-risk control group, the odds ratio for preterm delivery, PE and PIH were significantly higher for women with IgA nephropathy (Figure 3). 

Odds ratio (OR) for PE and PIH were particularly high: PE: OR 11.80; CI 7.53–18.48, PIH OR 10.39; CI 5.45–19.80. Conversely, the risk of preterm birth was about threefold that of the low-risk population (OR 3.37; CI 1.91–5.95), while the risk of neonates with low birth weight is about twice as high (OR 2.36; CI 1.52–3.66).

## 4. Discussion

This systematic review identified 13 cases series and 14 case reports, reporting on pregnancy-related outcomes in IgA nephropathy [27,28,29,30,31,32,33,34,35,36,37,38,39,40,41,42,43,44,45,46,47,48,49,50,51,52,53]. This is, at the best of our knowledge, the largest number of papers meta-analyzed or narratively discussed on IgA nephropathy in pregnancy, with particular attention to the risk of adverse pregnancy-related outcomes. In this regard, our review may add information to a previous recent systematic review, which included papers published since the start of Medline, and was mainly focused on the progression of kidney disease in patients with IgA nephropathy with and without pregnancy [23]. The review, that selected 4 papers with a control group, providing data of 273 patients with IgA nephropathy and of 241 patients with IgA nephropathy who did not become pregnant, supported a lack of disadvantage on the progression of nephropathy for IgA nephropathy patients with well-preserved function that became pregnant. 

The main novelty of the present review is to give more insight into the risk of pregnancy-related outcomes, that were already reported as frequent by the previous study, but that are meta-analyzed for the first time with respect to a low-risk population; in this context, limiting the analysis to the studies published since 2000 may allow for a better contextualization in the present panorama (Figure 3). 

The pattern of pregnancy-related outcomes is complex and the behavior of the risks may offer some interesting insights into the pathogenesis of pregnancy complications in IgA nephropathy. In fact, the meta-analysis identifies a particularly high risk of PE and PIH in women with IgA nephropathy, with an over ten-fold increase, within wide but consistent confidence intervals (Figure 3). In spite of these high odds ratios, the significantly increased risk of preterm birth was only about triple compared to the low-risk population and the incidence of newborns with low birth weight is only doubled, while no difference was found in the incidence of both caesarean sections and of small for gestational age babies (Figure 3). This finding suggests that the very high risk for hypertensive disorders of pregnancy is not accompanied by a correspondent increase of preterm babies, and of babies whose growth has been severely impaired, as it occurs in the “placental” early forms of preeclampsia, and therefore suggests that the related event occurs later, corresponding with the so-called “late” form of PE [54,55,56,57,58]. Such a form of PE is usually milder, and is often associated with maternal diseases, and indeed some authors distinguish between “maternal” and “placental” preeclampsia, the latter characterized by a primary defect of placentation, and frequently associated with intrauterine growth restriction. Conversely, the maternal form is more often of late onset, frequently, even if not always, less severe, and is associated with a lower risk of newborns that are small for gestational age, a pattern that may be consistent with the observations gathered in the present review [54,55,56,57,58,59]. 

With respect to disease progression, the lack of negative effect of pregnancy, at least in populations with relatively preserved kidney function, reported in the previous meta-analysis, is confirmed by our analysis, performed with a different, selection of the papers, and with the chance of adding several recent studies on this issue (Figure 2) [23]. In most of the studies retrieved by both reviews kidney function was normal or well-preserved before pregnancy and, in this context, the progression of the kidney disease does not appear to be affected by pregnancy. However, most studies fail to give information both on women with severe CKD and the duration of the observation time is not always clear. Furthermore, even if relatively long, the median follow-up period of 10 years described by Limardo, and of 4.5 years by Liu, could still be too short to draw conclusions, given the slow progression of early IgA nephropathy [31,36]. The interesting new suggestion that progression of kidney disease is limited to the cases who display adverse pregnancy outcomes should be confirmed in further studies [27].

The case reports, as expected, focus on exceptional events, and are probably affected by reporting and publishing biases. Despite these limits, they may underline that positive pregnancy outcomes are possible, even in severe advanced chronic kidney disease [40,41,42,43,44,45,46,47,48,49,50,51,52,53] (Table 5, Table 6 and Table 7). 

The major strength of this study is that it provides an updated systematic review in one of the most common nephropathies observed in young patients all over the world, gathering over 700 pregnancy published in the new millennium, and attempting, for the first time, a risk assessment with regard to the overall population. The integration with case reports, a further novel aspect of this review, underlines that a positive outcome is possible even in women with severe CKD, who are usually excluded from the larger case series. 

The main limitations of this review are linked to the heterogeneity of the studies in terms of populations, definitions and outcomes, as well as duration of follow-up. Heterogeneity is a common problem in systematic reviews on CKD pregnancies, which limits the value of the meta-analysis, and calls for a common language, a still unmet goal [60,61,62]. Furthermore, due to lack of data on low-risk populations, we employed the only available one, from a multicenter Italian study; the approach of choosing a reference population for a wider meta-analysis is not new, and was for example employed by Deshpande and coworkers in the pivotal analysis on pregnancy after kidney or after liver transplantation, which plotted the data against the reference USA population [63,64]. Such a choice is an obvious compromise, and the lack of matched populations limits the precision of the comparison; however, the differences with the low-risk populations are highly significant, and their pattern is consistent, thus allowing at least posing new hypotheses and indications for future research. 

Within these limits, we hope that our findings may be of interest for counseling and for tailoring clinical surveillance for clinicians working in obstetrics and in nephrology. 

Our study may also indicate uncovered fields for future research. First of all, more data is needed to better define the risk of adverse pregnancy outcomes, particularly so in case of kidney function decrease. Secondly, given the impossibility to clinically distinguish an increase in week and hypertension related to the IgA nephropathy from that related to preeclampsia, there is need for precise description of these cases, with stratification for previous hypertension, and univocal definitions of superimposed PE. In this regard, IgA nephropathy may represent a field for the systematic use of old and new biomarkers of PE and kidney disease [63,64,65,66,67,68]. 

## 5. Conclusions

Patients with IgA nephropathy and severe CKD should be informed about the paucity of data allowing a precise quantification of the risks. In patients with preserved kidney function, that represent the majority of those reported, the risk of progression of kidney disease is low, and may be limited to the cases who display adverse pregnancy related events. Conversely the risk of developing PE and PIH is very high compared to the low-risk population (OR > 10). The pattern of these hypertensive disorders of pregnancy, associated with a significant but milder increase in preterm delivery but not of small for gestational age babies, suggests the presence of late onset PE, in its “maternal” form, thought to affect fetal growth less severely than ‘placental’ early PE. This hypothesis, needing confirmation on a larger scale, may prove of interest in interpreting the increase in hypertensive disorders of pregnancy observed in chronic kidney disease.

## Figures and Tables

**Table 1:** IgA nephropathy: Main characteristics of the case series (at least 6 pregnancies).

Author Year [Ref]	Years	Country	Study	Objective, as Stated in the Study	Pregnancies (P) ^§§^ Women (W)
Park 2017 [27]	1979–2015	Korea	Ret	To assess the relationship between pregnancy and renal prognosis in women with IgAN and to investigate further whether obstetric complications are associated with renal prognosis	59 W 64 P 59 W controls (non-pregnant IgA)
O’Shaughnessy 2017 [28]	1996–2015	USA	Ret	To investigate the influence of glomerular disease subtype on pregnancy outcomes	17 W 18 P
Su 2017 [29]	2003–2014	China	Pro	To assess the effects of pregnancy on kidney disease progression and risk factors for adverse pregnancy outcomes in patients with IgAN	104 W 116 P 309 W controls (non-pregnant IgA)
Tocos 2017 [30]	2000–2016	Italy	Pro	To evaluate the maternofetal outcomes in different glomerulonephritis	27 W ^§^ 33 P 1418 P controls (low risk)
Liu 2014 [31]	2003–2012	China	Matched-cohort	To evaluate the safety of pregnancy in women with IgAN, as well as their risk factors for adverse pregnancy outcomes, as compared to non-pregnant women with IgAN	62 W 69 P 62 W controls (non-pregnant IgA)
Oh 2011 [32]	2004–2009	Korea	Ret	To investigate whether higher week at conception predicts a faster decline in maternal renal outcomes and to identify whether a week reduction prior to pregnancy attenuates the deterioration of postnatal maternal outcomes	52 W
Suetsugu 2011 (**) [33]	NR	Japan	Ret	To explore the clinical characteristics of predictive factors for hypertension in biopsy-proven IgA nephropathy patients with superimposed preeclampsia	34 W
Shimizu 2010 [34]	1995–2006	Japan	Pro	To evaluate the impact of the CKD staging in patients with IgAN on pregnancy and delivery	29 W 29 P 45 W controls (non-pregnant IgA)
Waness 2010 [35]	2000–2006	Saudi Arabia	Pro	To examine the natural history of pregnancies and their impact on renal function in Saudi females affected by IgAN	12 W 12 P
Limardo 2010 [36]	1974–2003	Italy	Ret multicenter cohort	To compare the long-term outcome of kidney disease in women with IgAN and preserved kidney function (sCr <1.2 mg/dL) who did and did not become pregnant. Data on 10 pregnant and 12 non pregnant women with sCr >1.2 mg/dL also gathered	136 W 229 P 87 W controls (non-pregnant IgA)
Donggyu 2010 (*) [37]	1987–2008	Korea	Ret	To clarify the influence of pregnancy on the natural course of IgAN	25 W 28 P
Ronkainen 2006 [38]	NR	Finland	Ret	To evaluate renal survival, morbidity, pregnancy complications and factors predicting outcome after childhood IgAN	10 W 22 P
Ronkainen 2002 [39]	NR	Finland	Ret	To assess long-term outcome of children with renal involvement at onset of Henoch-Schönlein purpura by comparison with those who have mainly extra-renal symptoms at referral	14 W 23 P
Overall number of women, pregnancies and controls					581 W 729 P ^§^ 562 non pregnant IgA controls 1418 low risk controls

CKD: chronic kidney disease; IgAN: IgA nephropathy; NR: not reported; P: pregnancy; Pro: prospective; Ret: retrospective; sCr: serum creatinine; W: woman. * Abstract only (congress proceeding); (**) paper in Japanese, abstract used. ^§^ not mentioned in article, additional information available in original database ^§§^ if number of pregnancies not specified, we assumed 1 pregnancy per woman.

**Table 2:** IgA nephropathy: maternal and fetal control policies, in the papers reporting on them (Case Series).

Author Year [Ref]	Control Policies
Su 2017 [29]	Follow up at least once a month before delivery, and every 1–6 months after delivery, with minimum follow up 12 months postpartum or until dialysis treatment
Tocos 2017 [30]	Follow up at least once monthly if week , hypertension or kidney function reduction
Liu 2014 [31]	Follow up every ≤1 month; eGFR decline; determination time-averaged MAP and week every 3 months
Shimizu 2010 [34]	BP, week , blood analysis and eGFR at the baseline at the time of detection of pregnancy; at 16, 22 and 30 weeks of pregnancy; at the time of delivery; and at 3 months and 1, 2 and 3 years after delivery
Waness 2010 [35]	Monthly measures of BP, 24 h week , sCr, CCr; close monitoring and follow up
Limardo 2010 [36]	Information gathered at time of biopsy and every 5 year thereafter: CCr, 24 h week , body weight, BP, therapy with ACEIs/ARBs or immunosuppressants
Donggyu 2010 (*) [37]	sCr followed up max 3 years after delivery

ACEI: ACE-inhibitor; ARB: angiotensin II-receptor blocker; BP: blood pressure; CCr: creatinine clearance; eGFR: estimated glomerular filtration rate, assessed by MDRD or EPI formula; IgAN: IgA nephropathy; MAP: mean arterial pressure; NR: not reported; sCr: serum creatinine; * Abstract only.

**Table 3:** IgA Nephropathy: Fetal and pregnancy outcomes in the case series.

Author Year [Ref]	All Cases Considered					Live Births Only		All Cases	All Cases, or As Stated
	P	Abort. Spont. Induced	Still Birth	Live Birth	Neo. Death	Preterm <37 weeks Early <34 weeks Extreme <28 weeks	NICU	PE-HT	Other
Park 2017 [27]	64	NR	NR	NR	NR	Preterm: 21 (33%) Early: 8 (13%)	NR	PE: 13 (20%)	LBW <2500 g: 16 (25%) LBW <1500 g: 6 (9%) SGA <10th: 6 (9%)
O’Shaughnessy 2017 [28]	18	NA	0	18 (100%)	2 (11%)	Preterm <37 w: 6 (33%), 5/6 induced/CS on maternal indication Preterm <32 w: 4 (22%)	NR	PE: 6 (33%)	Median GA: 37.5 week (36–39) Median BW: 2627 g (2136–3315) IUGR <10th: 2 (11%) IUGR<3rd: 0 Apgar 1 min: 8 (7–9) Apgar 5 min: 9 (9–9)
Su 2017 [29]	116	5 (4%) spont 2 (2%) induc	18 (16%)	90 (78%)	1 (1%)	Preterm: 13 (11%)	NR	GHT: 26/89 (29%) Severe PE: 12 (10%) PtU >3.5 g/day: 19/110 (17%)	CS: 62 (53%) Mean GA: 37.8 week ± 2.4 Mean BW: 3035 g ± 668 ^†^ LBW <2500 g: 16 (17%) ^†^ LBW <1500 g: 3 (3%) ^†^
Tocos 2017 [30]	33 cases	NA	NA	33 (100%)	NR	Preterm: 12 (36%) Early: 4 (12%) ^††^ Extreme: 1 (3%) ^††^	NR	GHT: 7/24 (29%) ^†††^ PE: 4/17 (24%) ^†††^	CS: 9 (27%) LBW: 10 (30%) SGA <10th: 4 (12%) ^††^ SGA <5th: 1 (3%) ^††^
	1418 controls	NA	NA	1418 (100%)	NR	Preterm: 89 (6%) Early: 13 (1%) Extreme: 2 (0.1%)	NR	HT: 66 (5%) PE: 25 (2%) PtU: 25 (2%)	CS: 379 (27%) GA: 39 weeks (25–42) BW: 3232 ± 476 g SGA <10th: 120 (8%) SGA <5th: 45 (3%)
Liu 2014 [31]	69	8 (12%) ^§^	2 (3%)	59 (86%)	NR	Preterm: 7 (10%)	NR	Severe PE: 6 W (10%)	CS: 42 (61%) LBW: 8/59 (14%) Mean BW: 2972 ± 654 g
Oh 2011 [32]	52	^§§^	NR	NR	NR	Preterm: 8 (15.4%)	4 (7.7%)	HT: <8 weeks 31 (60%)	CS: 24 (46.2%) LBW: 13 (25%)
Suetsugu 2011 (**) [33]	34	NR	NR	NR	1 (3%)	NR	NR	Superimp. PE: 13 (38.2%)	BW negatively correlated with glomerular sclerosis, sCr and BUN.
Shimizu 2010 [34]	29	0	0	29 (100%)	0	0 Gestation 38.0 ± 2 weeks	NR	No PE	CS: 5 (17.2%) BW: 2911.2 ± 138.7 g LBW: 0
Waness 2010 [35]	12	0	0	12 (100%)	NR	0	NR	HT: 12 (100%) PE: 3 (25%) HELLP: 1 (8.3%)	CS: 2 (HELLP and PE) BW: 3.1 kg LBW: 0 Apgar: normal (1’ and 5’)
Limardo 2010 [36]	229	15 spon 13 indu	5 (2.2%)	195 (85%)	1 (0.4%)	Preterm: 20 (10%)	NR	HT: 43/201 (21%) PE: 17 W (13%)	Mean BW: 3039 ± 610 g LBW: 22/195 (11%)
Donggyu 2010 (*) [37]	28	NR	NR	NR	NR	NR	NR	PE: 4 W (of 5 with sCr >2.0 mg/dL)	NR
Ronkainen 2006 [38]	22	At least 2 spont	NR	20	NR	Preterm: at least 6 (30%) Extreme: at least 2 (10%)	NR	HT: 10 (46%) Severe PE: 1 W (10%) PtU: 12 (55%)	6 (30%) of 20 live born infants from mothers with HT and/or week premature
Ronkainen 2002 [39]	23	NR	NR	NR	NR	NR	NR	HT and/or PtU: 16 (70%)	NR
Summary data	729	45/485 (9.3%)	25/473 (5.3%)	456/528 (86.3%)	5/426 (1.2%)	Preterm 95/608 (15.6%) Early: 18/135 (13.3%) Extreme: 3/53 (5.6%)	4/52 (7.7%)	PIH: 98/348 (28.2%) PE: 79/523 (15.1%)	CS: 144/311 (46.3%) LBW: 85/530 (16.0%) IUGR/SGA <10th: 12/115 (10.4%)

abort: abortions (<24 gestational weeks); W: women; BUN: blood urea nitrogen; BW: birth weight; CS: caesarean section; m: months; NR not reported; HELLP: hemolysis, elevated liver enzymes, low platelets syndrome; HT: hypertension; LBW: low birth weight (<2500 g); NA: not applicable, only included births >20 weeks or only live births; NICU: neonatal intensive care unit; NR: not reported; PE: preeclampsia; P: pregnancies; * Abstract only (**) paper in Japanese, abstract used, ^†^ data on 86/116 pregnancies, ^††^ not mentioned in article, additional information available in original database, ^†††^ calculated in W without baseline hypertension and/or week , ^§^ spontaneous abortion: 1 (2%); induced abortion: 1 (2%); embryo damage 3 (4%); fetal malformation 3 (4%)., ^§§^ 14 abortions in 80 women, not included in final analyses of the study. Note: if not otherwise clarified, numbers of cases with hypertension, PE and/or PtU were counted separately.

**Table 4:** IgA Nephropathy. Kidney function and other maternal outcomes in the case series reporting on them.

Author Year [Ref]	Age (years)	Kidney Function at Baseline	Other Maternal Outcomes and Main Results
Park 2017 [27]	28 (24–31) (cases)	eGFR: 80.0 (61.0–105.6) sCr: 0.90 mg/dL (0.70–1.00) PtU: 1.09 g/day (0.46–2.02) HT: 36 (61%)	Renal survival rate with gestational complications: 55.3% at 10 y; 46.1% at 20 years Renal survival rate without gestational complications: 97.3% at 10 y; 97.3% at 20 years Obstetric complications (PE, LBW and/or preterm birth), not pregnancy itself, associated with CKD progression, especially if eGFR <60, preexisting HT and PtU >1 g/day (all significant)
	26 (23–32) (controls)	eGFR: 85.0 (64.7–102.0) sCr: 0.80 mg/dL (0.70–1.00) PtU: 0.87 g/day (0.43–1.60) HT: 33 (56%)	Renal survival rate: 80.3% at 10 years; 70.4% at 20 years
O’Shaughnessy 2017 [28]	31.3 (23.0–33.8)	eGFR: 72 (61–90) (9/18 P) sCr: 1.0 mg/dL (0.8–1.2) (9/18 P) PtU spot: 1.3 g (0.9–4.1) (8/18 P)	≥200% increase PtU (2–12 m postpartum): 2/6 (33.3%) ≥150% increase sCr (2–12 m postpartum): 1/8 (12.5%) ESRD 1 year postpartum: 2 (11.1%) ^§^ Active IgAN during pregnancy: 12 (66.7%). Dialysis during pregnancy: 0
Su 2017 [29]	27.2 ± 3.5 (cases)	eGFR: 102.6 ± 23.9 PtU: 1.04 g/day (0.03–7.25) HT: 15 (14%) Follow up: 67 ± 34 months	Persistent HT postpartum: 12/89 (13%). Irreversible PtU worsening: 7 (6%) PtU at pregnancy start or first trimester: risk factor for severe PE and infant loss ESRD: 4 (4%) ^§^ ESRD/>50% decrease eGFR: 7 (7%) Significant decrease kidney function after pregnancy in CKD stage 3–4 only
	28.7 ± 6.3 (controls)	eGFR: 94.5 ± 26.7 PtU: 1.29 g/day (0.02–11.78) HT: 52 (17%) Follow up: 65 ± 34 months	ESRD: 18 (6%) ^§^ ESRD/>50% decrease eGFR: 31 (10%)
Tocos 2017 [30]	31.9 ± 5.2 (cases)	eGFR: 89.9 ± 32.7 sCr: 0.87 mg/dL (0.50–2.88) PtU ≥0.5 g/day: 13 (41%) HT: 9 (27%)	Worsening CKD stage during pregnancy: 1/33 (3%) ^§§^ Increased risk of PE but not of preterm delivery suggests late maternal PE
	31.2 ± 5.5 (controls)	HT: none	
Liu 2014 [31]	27.3 ± 3.6 (cases)	eGFR: 102.3 ± 21.9 PtU: 1.27 (0.06–7.25) g/day HT: 7 (11%)	HT after pregnancy 8 (13%); MAP during follow up 86.4 ± 8.6 Kidney disease progression: 4 (6%); decrease eGFR >50%: 3 (5%); ESRD: 1 (2%) ^§^ Mean change eGFR: −2.5 mL/min (−6.7 to 0.06) PtU during follow up: 0.67 g/day (0.10–6.72) Proteinuria in pregnancy borderline significant for adverse pregnancy outcomes
	27.8 ± 4.4 (controls)	eGFR: 103.4 ± 20.8 PtU: 1.09 (0.06–8.37) g/day HT: 4 (6%)	MAP during follow up 85.4 ± 7.3; Kidney disease progression: 6 (10%) decrease eGFR >50%: 4 (7%); ESRD: 2 (3%) ^§^ Mean change eGFR −2.4 –−7.1 to 2.4) mL/min PtU during follow up: 0.68 (0.07–4.30) g/day
Oh 2011 [32]	30.5 (25.0–39.0)	eGFR: 91.2 (24.1–157.0) mL/min MAP: 89.6–99.3 mmHg	eGFR after delivery 77.8 (19.8–150.0) Median ΔGFR with ≤30% reduction week prior to conception: 13% Median ΔGFR with >30% reduction week prior to conception: 8.7% MAP during pregnancy 96.7–102 Significant increase sCr (0.8–1.0 mg/dL) and PtU (0.7–1.5 g/g) after delivery
Suetsugu 2011 (*) [33]	NR	NR	Superimposed PE: preconception SBP, sCr, BUN higher; CCr and eGFR lower Delivery: sCr, BUN, uric acid higher; CCr and eGFR lower (significant) At delivery correlation between BP and histological severity, week and sCr
Shimizu 2010 [34]	27.3 ± 4.0 (cases)	eGFR mL/min CKD1: 97.3 ± 9.4 CKD2: 74.1 ± 4.5 CKD3: 54.4 ± 11.6	eGFR 3 year after delivery (mL/min): CKD1: 93.0 ± 1.6; CKD2: 78.2 ± 11.8; CKD3: 58.5 ± 14.4; Overall: baseline 68.9 ± 14.4—three years after 68.5 ± 14.9 sCr baseline—3 year after delivery (mg/dL): 1: 0.68–0.64; 2: 0.75–0.72; 3:0.94–0.90. Overall: 0.83 ± 0.20–0.75 ± 0.14 PtU baseline—3 year after delivery (g/day): CKD1: 0.19 ± 0.1–0.20 ± 0.28; CKD2: 0.39 ± 0.22–0.48 ± 0.44; CKD3: 0.77 ± 0.31–0.38 ± 0.33 (**) BP constant in all CKD groups
	28.1 ± 5.1 (controls)	eGFR: 70.9 ± 20.7	eGFR after 3 years (mL/min): 68.6 ± 14.4 sCr baseline—after 3 years (mg/dL): 0.8 ± 0.15–0.88 ± 0.16. PtU baseline—after 3 years (g/day): 0.85 ± 0.65–0.40 ± 0.26 No new onset hypertension
Waness 2010 [35]	28.6	CCr 88.6 mL/min sCr: 0.99 mg/dL BP: 128.2/82.1 mmHg PtU 535.2 mg/day	In 3rd trimester: BP 163.7/90.3 mmHg PtU 2179.2 mg/day CCr 77.4 mL/mins Cr 84.3 mmol/L
Limardo 2010 [36]	26.72 ± 4.27 (cases)	sCr 0.87 ± 0.15 CCr 92 ± 17 PtU 1.0 (0–6) g/day HT: 27 (20%)	After 10 years: 36% on steroids and/or immuno-depressors; 61% on ACEI or ARBs Significant CCr decrease (−1.2 mL/min/year) in women with PtU >1 g/day at diagnosis, not modified by number of pregnancies, hypertension, PE Doubling of sCr in 13 (9.6%); start of dialysis in 3 (3.4%) ^§^; new-onset HT in 34 (31%) of 109 previously normotensive women
	26.19 ± 5.15 (controls)	sCr 0.86 ± 0.16 CCr 89 ± 18 PtU 0.5 (0–7.6) g/day HT: 10 (11%)	After 10 years: 29% on steroids and/or immune-depressors; 47% on ACEI or ARBs Doubling of sCr in 7 (8%); start of dialysis in 2 (1.5%) §; new-onset HT in 16 of 77 (21%) previously normotensive women
Donggyu 2010 * [37]	NR	NR	PE in 4 of 5 women with sCr >2.0 mg/dL at delivery ESRD within 2 years in 2/2 W with sCr >2.5 mg/dL in postpartum All women with sCr <2.5 mg/dL in postpartum had stable sCr 3 year after delivery
Ronkainen 2006 [38]	NR	NR	ESRD 2.6 year after delivery in 1 hypertensive woman with slightly impaired renal function before pregnancy ^§^
Ronkainen 2002 [39]	NR	NR	HT or PtU in pregnancy: 9 (64.3%), of whom 5 reported poor outcome (not specified); no poor outcome reported in women without HT or PtU in pregnancy
Summary data baseline	GFR or CCr >100 mL/min in 2/9 study reporting on this item GFR <100 mL/min in 7/9 studies PtU ≥0.5 g/day in 6/7; <0.5 g /day in 1/7 studies (**) Hypertension in 11–61% in 4 studies, in other not clearly defined at baseline		
Summary data progression	ESRD: 11/330 (3.3%) cases vs 22/458 (4.8%) controls, reported on by 5 studies of whom 3 provided a control group ^§^ Park: no significant difference between cases and non-pregnant controls. Significant better renal survival in cases without vs with obstetric complications, and in cases without obstetric complications vs non pregnant controls Su: no significant difference in incidence ESRD or eGFR decrease between cases and non-pregnant controls Liu: no difference between cases and non-pregnant controls over follow-up Shimizu: no difference in eGFR decrease between pregnancy and non-pregnancy Limardo: no significant difference in all outcomes (start of dialysis, doubling of serum creatinine, new onset hypertension)		

BP: blood pressure; CCr: creatinine clearance; (e)GFR: (estimated) glomerular filtration rate; ESRD: end-stage renal disease; HT: hypertension; MAP: mean arterial pressure; NR: not reported; P: pregnancies; PE: preeclampsia; week sCr: serum creatinine. * Abstract only (*) article in Japanese, abstract used. ** discrepant data between the various CKD stages and overall, as for week : Overall: 0.86 ± 0.80–0.56 ± 0.48. ^§^ used in meta-analysis despite of difference in follow-up (1 to 10 years); ^§§^ not mentioned in article, additional information available in original database.

**Table 5:** IgA Nephropathy. Case reports: baseline data.

Author Year [Ref]	Country	Age (years)	sCr-GFR-PtU	Other Data at Referral	Main Drugs in Pregnancy
Kaul 2016 * [40]	India	NR	NR	IgAN new-onset	Steroids, fish oil
		NR	NR	IgAN new-onset	Steroids, fish oil
		NR	NR	IgAN new-onset	Steroids, fish oil
Lim 2016 * [41]	USA	NR	NR	IgAN (diagnosed several years postpartum)	NR
Sun 2015 [42]	China	26	PtU 1–2+	IgAN new-onset	NR
Nagai 2015 [43]	Japan	37	PtU postpartum	HSPN postpartum	NR
Liang 2015 * [44]	USA	32	PtU 2 g/day	IgAN new-onset	NR
Zand 2014 [45]	USA	18	sCr 1.8 mg/dL	IgAN	NR
Cornelis 2013 [46]	The Netherlands	21	CCr 20–25 mL/min	IgAN	Methyldopa, labetalol, EPO, thyroid hormones, oral iron
Hou 2013 [47]	USA	28	CCr 79 mL/min PtU 1.13 g/day	IgAN new-onset	Methyldopa, labetalol, hydralazine, magnesium (31 week), steroid prophylaxis (31 week)
Goifrè 2007 [48]	Italy	25	sCr 2.2 mg/dL PtU 1 g/day	IgAN	ASA, oral iron, vitamins, vaginal dinoprostone gel (36 week)
		30	sCr 8 mg/dL		EPO, vit D, calcium carbonate, ritodrin (29 week)
		32	HD		EPO, vit D, calcium carbonate, ritodrin (30 week)
Tanno 2007 [49]	Japan	31	sCr 0.8 mg/dL	HSPN recurrence in renal allograft	Methyldopa, amlodipine Immunosuppressors (not clear)
Barquero-Romero 2006 [50]	Spain	36	sCr 0.50 mg/dL PtU 1+	HSPN new-onset	Methylprednisolone
Koizumi 2004 [51]	Japan	30	PtU +	HSPN new-onset	Low dose oral steroids for 3 week
Cusi 2003 [52]	Italy	29	sCr 1.5 mg/dL PtU 1.2 g/day	IgAN	Methyldopa, nifedipine, clonidine, EPO, steroid prophylaxis (26 week)
Amir 2002 (*) [53]	Saudi Arabia	NR	sCr 2.7 mg/dL PtU 5.4 g/day	IgAN with P-ANCA	Cyclophosphamide, prednisone
Summary data		28.8 (18–37)	sCr <1.0 mg/dL: 2/9 (22.2%) sCr ≥1.0 mg/dL: 5/9 (55.6%) CCr <90 mL/min: 2/9 (22.2%) CCr ≥90 mL/min: 0 PtU >= 0.5 g/day: 5/5 (100%) reporting quantitatively	IgAN: 12 (new-onset: 6) HSPN: 4 (new-onset: 3)	Antihypertensive agent: 4/10 Immunosuppressors: 4/10

ASA: acetyl salicylic acid; CCr: creatinine clearance; EPO: Erythropoietin; GFR: Glomerular filtration rate; h: hours; HSPN: Henoch-Schönlein purpura nephropathy; IgAN: IgA nephropathy; i.v.: intravenous; methyldopa: alpha-methyldopa; NR: Not reported; week vit: vitamin; w: week. * Abstract only (*) abstract used.

**Table 6:** IgA Nephropathy. Case reports: fetal outcomes.

Author Year [Ref]	Pts	GW	Parity	Delivery	Indication for Delivery	NICU	APGAR 1–5 min Infant Outcomes	Sex	Weight (g)	Centile *
Kaul 2016 * [40]	3	NR	NR	NR	NR	NR	All live births	NR	NR	NR
Lim 2016 * [41]	1	NR	NR	NR	NR	NR	NR	NR	NR	NR
Sun 2015 [42]	1	40	Primi	CS	NR	NR	NR	NR	NR	NR
Nagai 2015 [43]	1	At term	NR	Vaginal	None	NR	NR	NR	NR	NR
Liang 2015 * [44]	1	36 + 5	Gravi 7 Para 1 P1051	Vaginal induced	Presumed superimposed PE	NR	NR	F	NR	NR
Zand 2014 [45]	1	32	NR	Vaginal	None	NR	Healthy	M	NR	NR
Cornelis 2013 [46]	1	36	Primi	Vaginal assisted	Sudden HT	YES	9 and 10 Wet lung syndrome, NICU non-invasive ventilatory support 1 day. Discharged 8 days later.	M	2480	25
Hou 2013 [47]	1	31	Gravi 2 Para 0	CS	PE, failed induction	NR	3 and 8 Normal development 11 years later.	F	1596	64
Goifrè 2007 [48]	1	38	Gravi 1 Para 0	Vaginal	None	YES	8 and 9 NICU, discharged 20 days later.	M	3150	45
		33	Gravi 2 Para 1	CS	NR	YES	7 and 8 NICU for RD (ventilatory support for 6 h); discharged 20 days later.	M	2190	65
		33	Gravi 3 Para 2	CS	NR	YES	5 and 8 NICU for RD (ventilatory support for 2 days); discharged 30 days later.	M	2500	90
Tanno 2007 [49]	1	28	NR	CS	Impaired umbilical flow and fetal growth	NR	No obvious anomalies	NR	999	39 **
Barquero-Romero 2006 [50]	1	39	Multi	Vaginal	None	NR	Healthy at 3 m follow up	M	3380	41
Koizumi 2004 [51]	1	40	Primi	Vaginal	None	NR	Healthy	M	2986	11
Cusi 2003 [52]	1	31	NR	Vaginal	None	NR	8 and 9 Healthy	F	1626	68
Amir 2002 (*) [53]	1	NR	NR	NR	NR	NR	NR	NR	NR	NR
Summary data	16 (18 cases)	<37 w: 8/13 (61.5%) ≥37 w: 5/13 (38.5%)	Primi: 4/9 (44.4%) Multi: 5/9 (55.6%)	Vaginal: 8/13 (61.5%) CS: 5/13 (38.5%)	Maternal complications: 3/10 (30%) Fetal complications: 1/10 (10%) None: 6/10 (60%)	All reported cases had favorable outcomes; NICU reported in 4 cases		F: 3/10 (30%) M: 7/10 (70%)	<2500 g: 5/9 (55.6%); <1500 g: 1/9 (11.1%)	AGA: 9/9 (100%) (calculated upon INeS charts)

AGA: appropriate for gestational age; CS: cesarean; d: days; F: female; Gravi: gravidity; GW: Week of gestation; h: hours; HT: hypertension; LGA: large for gestational age; M: male; m: months; Multi: multipara; NICU: neonatal intensive care unit; NR: not reported; Para/P: parity; PE: preeclampsia; Primi: primipara; Pts: Patients; RD: respiratory distress; w: weeks. * Abstract only (*) abstract used * Centiles calculated according to the INeS charts (reference). If parity or sex are not specified: male sex and primipara considered.

**Table 7:** IgA Nephropathy. Case reports: maternal outcomes.

Author Year [Ref]	PE/Other	Maternal Outcomes, as Reported in the Paper
Kaul 2016 * [40]	NR	All 3 patients treated with steroids and fish oil, complete remission in all 3 patients
Lim 2016 * [41]	HT and PtU at 22 weeks, presumed PE. No follow up postpartum.	Several years postpartum (age 25) presentation with severe HT and cardiac failure, pulmonary edema, hematuria, week , small hypo-echoic kidneys on ultrasound. Kidney biopsy: IgAN with severe atrophy and fibrosis
Sun 2015 [42]	None	5 days postpartum atypical hemolytic uremic syndrome (AKI, nephrotic syndrome, thrombocytopenia and hemolytic anemia), HD for 5 weeks
Nagai 2015 [43]	6th month HSP purpura, during pregnancy normal urinalysis	1 m postpartum HSPN without renal dysfunction, and anti-PL-7 anti-synthetase syndrome with interstitial lung disease and subclinical myopathy
Liang 2015 * [44]	HT, week and hematuria	Normalization BP; persistent PtU and hematuria; biopsy proven IgAN 1.5 years later
Zand 2014 [45]	HT, anemia, atypical hemolytic uremic syndrome, start HD	On HD; living kidney donor transplant 5 months later
Cornelis 2013 [46]	26 week start intensive HD for rapidly progressive deterioration kidney function; sudden HT 35 + 5 weeks	2 weeks postpartum restart HD; 1 year later living-donor kidney transplant
Hou 2013 [47]	HT, PE 31 weeks	1 year later deterioration kidney function; 11 years later evaluation for kidney transplant
Goifrè 2007 [48]	Anemia	Pre HD (CKD in 1st pregnancy)
	Anemia; start HD end 1st trimester; polyhydramnios 28 weeks	On HD
	Anemia, polyhydramnios 30 weeks	On HD; 1 year later: kidney transplant
Tanno 2007 [49]	17 week HT and PtU, microhematuria; worsening of kidney function at 24 weeks and 28 weeks	Postpartum decrease BP and sCr, PtU from 6.0 to 1.0 g/day 6 months postpartum kidney biopsy: HSPN recurrence in renal allograft with additional focal segmental membranous nephropathy with C1q deposition
Barquero-Romero 2006 [50]	HSP at 36 weeks, good response to steroid	Healthy at 3 months follow up
Koizumi 2004 [51]	Elevated levels CRP and ALT/AST	Normalization of blood analysis and urinanalysis
Cusi 2003 [52]	HT, anemia	Persistence of HT and anemia
Amir 2002 (*) [53]	11 week rapidly progressive GN: sCr 2.7 mg/dL, PtU 5.4 g/24 h	Good response to cyclophosphamide and prednisone: sCr 1.4 mg/dL, PtU 0.516 g/day, 18 months after diagnosis no significant clinical problems and stable kidney function
Summary data	PE: 1 Pregnancy induced HT: 6 Start HD in pregnancy: 3	Different outcomes of the kidney function also depending upon the disease

AKI: acute kidney injury; BP: blood pressure; CRP: C-reactive protein; d: days; GN: glomerulonephritis; HD: hemodialysis; HT: hypertension; HSPN: Henoch-Schönlein purpura nephropathy; IgAN: IgA nephropathy; NR: Not reported; PE: preeclampsia; week * Abstract only (*) abstract used.
